# Nutritional Deficiencies Before and After Bariatric Surgery in Low- and High-Income Countries: Prevention and Treatment

**DOI:** 10.7759/cureus.55062

**Published:** 2024-02-27

**Authors:** Faiza A Kamal, Lucas Y Fernet, Miguel Rodriguez, Fatima Kamal, Naofal K Da Silva, Omar A Kamal, Alberto Ayala Aguilar, Victor S Arruarana, Marily Martinez Ramirez

**Affiliations:** 1 General Practice, University of Nottingham, Nottingham, GBR; 2 Surgery, Universidad de Oriente, Houston, USA; 3 Internal Medicine, Connecticut Institute for Communities, Danbury, USA; 4 Department of Surgery, Baptist Hospital of Miami, Miami, USA; 5 Biological Sciences, University of Alberta, Edmonton, CAN; 6 General Practice, Universidad del Noreste, Tampico, MEX; 7 Internal Medicine, Brookdale University Hospital Medical Center, New York, USA; 8 Internal Medicine, Universidad Nacional Autonoma de Mexico, Mexico City, MEX

**Keywords:** high income, low income, bariatric, nutritional deficiencies, bariatric surgery

## Abstract

Nutritional deficiencies represent a prevalent concern among individuals with obesity, stemming from suboptimal dietary habits, chronic inflammation, and preoperative weight reduction efforts. Bariatric surgical interventions, employing either restrictive, malabsorptive or a combination of the two methods, further compound these deficiencies. Commonly observed nutritional deficits following bariatric surgeries include vitamin B_12_, vitamin D, thiamine, folate, iron, and protein deficiencies. These deficiencies are further complicated by disparities in healthcare resources and income that distinguish low, medium, and high-income countries. The escalating rates of obesity in low- and medium-income countries are primarily attributed to the increasing availability of cheap, nutritionally depleted, and processed foods, coupled with limited access to healthcare. The provision of bariatric surgical interventions in such regions is hindered by the lack of appropriately trained medical personnel and adequate infrastructure. Additionally, the crucial facets of postoperative care, including diligent follow-up, precise weight loss monitoring, and the administration of appropriate nutritional supplements, often remain lacking. This narrative review provides a comprehensive examination of the prevention and treatment of nutritional deficiencies before and after bariatric surgery in the context of varying healthcare resources and income levels. Bariatric procedures and their global prevalence are discussed, and the prevalence, symptoms, and management strategies of specific nutritional deficiencies are explained. This review also outlines practical strategies for providing more equitable care in low- and medium-income countries.

## Introduction and background

Bariatric surgery stands as a cornerstone in the treatment of obesity, offering transformative weight loss and addressing associated comorbidities such as diabetes and cardiovascular disease [1]. Despite its success, it carries a notable risk of inducing patient vitamin and mineral deficiencies. Among the most prevalent deficiencies reported in bariatric surgery patients are vitamin D, B12, folate, and iron [2]. Baseline preoperative deficiencies are robust predictors of postoperative nutritional deficits, underscoring the need to identify and rectify preoperative deficiencies to mitigate potential long-term complications [2]. Patient non-compliance with prescribed supplements exacerbate these deficiencies; a proactive approach that combines education, counseling, monitoring, and support is essential to combat this issue. By empowering patients to prioritize their nutritional needs, healthcare providers can help optimize long-term outcomes and minimize complications postoperatively.

Amidst these clinical considerations, disparities in healthcare resources and income levels between low, medium, and high-income countries emerge as barriers to achieving global health equity. These disparities span socioeconomic, political, ethnic, and cultural dimensions. While high-income nations tout universal access to healthcare, disparities in health outcomes persist, intricately linked to income disparities and other socioeconomic determinants. Conversely, low- and medium-income countries (LMICs) confront pervasive obstacles to healthcare access, including resource scarcity and infrastructural limitations [3]. In light of these complex healthcare landscapes, this review explores the importance of addressing nutritional deficiencies before and after bariatric surgery, contextualized within the distinct healthcare resources and income levels of low, medium, and high-income countries. Through this comparative analysis, we aim to delineate actionable strategies for preventing and managing nutritional deficiencies, thereby contributing to advancing global health equity within the realm of bariatric surgery.

## Review

Overview of bariatric surgeries

Bariatric procedures are offered to patients who meet specific criteria aimed at reducing morbid obesity and its associated health risks. According to the American Society for Metabolic and Bariatric Surgery (ASMBS), indications for metabolic and bariatric surgery include patients with a body mass index (BMI) greater than or equal to 35 kg/m2, irrespective of the presence, absence, or severity of comorbidities, and patients with a BMI greater than 30 kg/m2 who cannot achieve substantial weight loss or improvement in comorbidities, such as type 2 diabetes or hypertension, with non-surgical methods [4]. 

Bariatric surgical procedures promote weight loss through three mechanisms: malabsorptive, restrictive, and a combination of the two [5]. Malabsorptive procedures, such as jejunoileal bypass and biliopancreatic diversion, decrease the surface area for nutrient absorption in the small intestine or divert food away from the biliopancreatic secretions, which decreases their absorption [5-7]. Malabsorptive procedures have a higher incidence of postoperative nutritional deficiencies [8]. Restrictive procedures involve reducing caloric intake by altering the stomach capacity. This can be achieved through techniques such as resection, bypass, or creation of a proximal gastric outlet. Vertical banded gastroplasty and laparoscopic adjustable gastric banding are restrictive procedures that involve placing a gastric band around the stomach to restrict food intake and promote early satiety. These procedures have largely been replaced by sleeve gastrectomy, where most of the stomach is surgically resected, leaving a smaller “sleeve” to hold the food. Roux-en-Y gastric bypass (RYGB) surgery is renowned for its dual mechanism of action. This procedure involves the creation of a small gastric outlet to limit food intake. It also involves intricate anatomical alterations that result in the diversion of food past a significant portion of the small intestine, decreasing nutrient absorption. The hormonal changes elicited by this procedure also facilitate weight loss by impacting hunger regulation through cellular communication [6,9,10].

Several studies have found bariatric surgery to be superior to non-surgical methods for weight loss in obese patients. There is evidence that, compared to non-surgical treatments, bariatric surgery results in greater weight loss and higher rates of remission of type 2 diabetes and metabolic syndromes [11]. Patients who had undergone bariatric surgery also reported more significant improvement in their quality of life and further reduction in their use of antidiabetic, antihypertensive, and lipid-lowering agents compared to patients choosing non-surgical weight loss methods [11].

Global prevalence of obesity and bariatric surgeries

According to the World Health Organization (WHO), the prevalence of obesity worldwide has tripled since 1975, with more than 650 million adults being obese in 2016 [12]. Obesity, while known to be prevalent in high-income countries, has also become a common health concern in LMICs. Low-income countries are defined as those with a gross national income (GNI) per capita of $1,135 or less, middle-income countries have a GNI per capita of $1,136 - $13,845, and high-income countries are those with a GNI per capita of more than $13,846 [13].

Obesity rates in LMICs such as Tonga (77.1%), Tuvalu (62.2%), and Mexico (36.9%) are higher than in many high-income countries such as the United States (42.7%), Australia (31.7%), Canada (24.3%) or France (17%) [14]. The rise in the prevalence of obesity in LMICs is multifactorial. A significant contributor to this is the increased availability of nutrient-depleted, calorie-dense foods that are high in fat, sugar, and salt, which are cheaper alternatives to fresh, healthy foods [15-18]. Furthermore, globally, manual labor jobs have transitioned to more sedentary, office-based jobs, decreasing levels of physical activity; similarly, the introduction of motorized public transport has reduced the need for walking and cycling [15]. Even leisure time worldwide has shifted away from sports and physical activities to sedentary behaviors such as watching television. Chronic stress associated with poverty, unemployment, crime, and financial insecurity, is also likely to contribute to rising obesity through consistently elevated cortisol levels and increased appetite [19]. In contrast with high-income countries, however, the management of obesity in LMICs is particularly challenging due to lack of resources, finances, and medical/surgical training. Finally, in some cultures, particularly in sub-Saharan Africa, obesity is regarded as desirable and is associated with beauty, wealth, and higher social status. On the other hand, thin individuals are stigmatized and are associated with HIV/AIDS (human immunodeficiency virus/acquired immunodeficiency syndrome). Consequently, people from these cultures are more reluctant to engage in weight loss programs [15,20]. 

**Figure 1:**
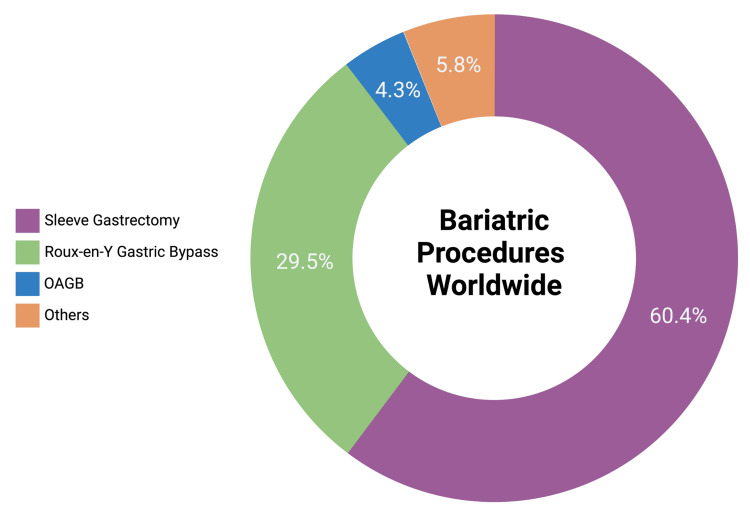
Most common bariatric surgeries performed worldwide 2021-2022. Data represents 480,970 procedures performed across 25 countries [23] OAGB = One-Anastomosis Gastric Bypass Image credits: Omar A. Kamal, 2024 (created with BioRender.com)

Bariatric surgery is becoming an increasingly prevalent procedure, with more than 579,000 surgeries performed worldwide in 2014 [21]. Growing rates of obesity, and drastic reduction in the rates of surgical complications (from 11.7% to 1.4%) and mortality (from 1% to 0.04%) from 1998 to 2016, have contributed to the growing demand of bariatric surgery worldwide [22]. According to the International Federation for the Surgery of Obesity and Metabolic Disorders (IFSO), some LMICs such as Brazil (70,490 procedures in 2021-2022), China (30,071), Mexico (7,058) and Iran (6,772) contribute significantly to the global total of bariatric procedures, alongside high-income countries such as the United States of America (209,527), France (38,890) and Australia (20,222) [23]. Data from 480,970 procedures performed across 25 countries showed that sleeve gastrectomy was the most common bariatric procedure worldwide (60.4%), followed by Roux-en-Y gastric bypass (29.5%) and one anastomosis gastric bypass (4.3%) (see Figure 1) [23].

Nutritional deficiencies before and after bariatric surgeries

Nutritional deficiencies affect both obese and non-obese individuals, with a higher risk associated with extremes of BMI [24]. Factors increasing this susceptibility include poor dietary choices, such as energy-dense foods lacking essential micronutrients [25-27], chronic inflammatory processes associated with obesity, which hinder micronutrient absorption, such as hepcidin secretion causing iron deficiency [28], and hypocaloric diets followed to achieve weight loss preoperatively, which provide insufficient micronutrients [2].

**Figure 2:**
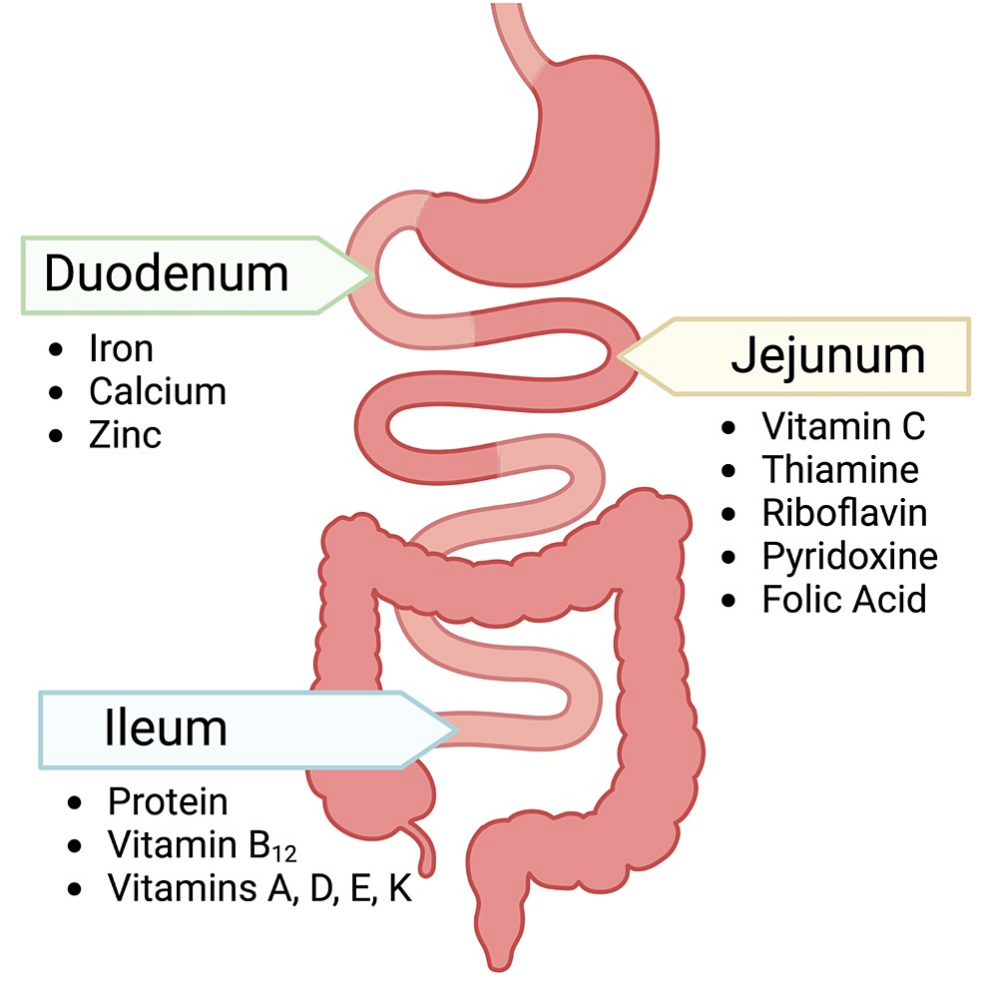
Sites of absorption of different micronutrients and proteins in the small intestine. Image credits: Omar A. Kamal, 2024 (created with BioRender.com).

Bariatric procedures disrupt gastrointestinal anatomy and physiology, increasing vulnerability to postoperative nutritional complications. Different micronutrients are absorbed in specific parts of the small intestine (see Figure 2), and therefore, postoperative micronutrient deficiencies can vary with the type of procedure performed. Caloric intake reduction is inevitable, averaging approximately 950 fewer kilocalories per day post-gastric sleeve surgery [29], further enabling deficiencies. Protein malabsorption is a major macronutrient deficiency observed in bariatric surgery patients [30]. Altered physiology impairs protein intake, digestion, and absorption. Protein malnutrition may also result from poor oral intake due to vomiting or maladaptive eating behaviors postoperatively [31], presenting as edema, hearing loss, and low serum albumin.

**Figure 3:**
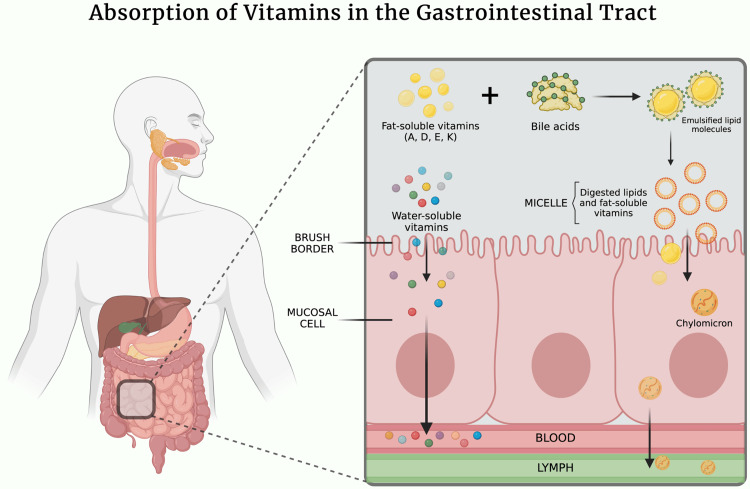
Mechanism of absorption of fat-soluble and water-soluble vitamins. Image credits: Omar A. Kamal, 2024 (created with BioRender.com)

Vitamin D deficiency, although common in the general population, has a higher prevalence in obese patients [32], reaching up to 94% [28]. Post-bariatric surgery, 10% to 73% of patients develop vitamin D deficiency. Fat-soluble vitamins (A, D, E, and K) require bile acids and pancreatic enzymes in order to emulsify and form micelles, which allow their absorption by the brush border epithelium of the small intestine. Once absorbed, these vitamins and dietary fats form part of the chylomicrons which are secreted into the lymphatic system (see Figure 3). This sequence of events can be disrupted by bariatric surgery, leading to reduced absorption and ultimately, deficiency [25,33]. Although commonly asymptomatic, vitamin D deficiency can lead to decreased calcium absorption, with secondary hyperparathyroidism and bone resorption as a result, ultimately resulting in fragility fractures.

Iron deficiency occurs in 33-49% of patients within two years post-bariatric surgery [31], particularly in procedures bypassing the duodenum and proximal jejunum (see Figure 2). Symptoms include microcytic anemia, fatigue, and lethargy. Vitamin B12 deficiency, especially in RYGB [34], may manifest after three years of inadequate intake, causing megaloblastic anemia and neuropathy [30]. The absorption of water-soluble vitamins is facilitated by carrier-mediated mechanisms [33]. Common deficiencies include thiamine (up to 49%) [35], causing nausea, constipation, and Wernicke-Korsakoff syndrome, and vitamin C (up to 34.6%) leading to scurvy [30]. Deficiencies in essential minerals such as magnesium, zinc, copper, and selenium have been reported in bariatric patients. These deficiencies impact various biochemical pathways and can cause neurological, cardiac, and gastrointestinal issues. Table 1 details micronutrient deficiencies, their prevalence preoperatively and incidence postoperatively, signs, symptoms, and management.

**Table 1:** Summary of micronutrient deficiencies observed in patients after bariatric surgeries. * Only to be used as a guide – treatment should ultimately be based on clinical context and local protocols. LAGB: laparoscopic adjustable gastric banding; RYGB: Roux-en-Y gastric bypass; SG: sleeve gastrectomy; BPD/DS: biliopancreatic diversion/duodenal switch; IU: international units; IM: intramuscular; SQ: subcutaneous

Micronutrient	Preoperative prevalence	Postoperative incidence	Symptoms	Recommended doses for micronutrient supplementation by surgery type *	
				Supplementation to prevent deficiency	Treatment for established deficiency
Vitamin A [27,36-39]	17%	Up to 70%	Night blindness, xerophthalmia, keratomalacia, xerosis cutis	LAGB: 5000 IU/day RYGB and SG: 5000 – 10,000 IU/day DS: 10,000 IU/day	Corneal changes absent: 10,000 – 25,000 IU/day until clinical improvement Corneal changes present: 50,000 – 100,000 IU/day given IM for 3 days, followed by 50,000 IU/day IM for 2 weeks
Vitamin D [2,25,36-40]	20.4 - 97.5%	10-73%	Commonly asymptomatic, but can present as osteomalacia with bone pains, fragility fractures and signs of hypocalcemia	Vitamin D_3_: 3000 IU/day until blood levels of 25-hydroxyvitamin D levels exceed 30 ng/mL	Vitamin D_3 _(preferred): 3000 – 6000 IU/day Vitamin D_2_: 50,000 IU 1-3 times/week
Vitamin E [37-41]	Rare	Rare	Ataxia, hemolytic anemia, peripheral neuropathy	15 mg/day regardless of surgery type	Potential benefits seen with 100 – 400 IU/day but optimal dose not yet defined (37). Some evidence that 500 – 1000 IU/day may be beneficial for replenishment
Vitamin K [36,37,39]	Not widely reported	Uncommon	Signs of hemorrhage	LAGB: 90 – 120 mg/day RYGB and SG: 90 – 120 mg/day DS: 300 mg/day	Acute malabsorption: parenteral 10 mg Chronic malabsorption: either 1 – 2 mg/day orally or 1 – 2 mg/week parenterally
Vitamin C (Ascorbic Acid) [24,30,36,35]	36%	34.6%	Scurvy: swollen gums, mucosal bleeding, arthralgias, anemia, impaired wound healing	No specific dosing post-bariatric surgery has been established. Take as part of multivitamin tablets (take 1-2 tablets/day)	200 mg/day
Vitamin B_1_ (Thiamine) [27,35-40]	15-29%	49%	Dry Beriberi: peripheral neuropathy, paralysis, confusion Wet Beriberi: high output cardiac failure, oedema, dilated cardiomyopathy Wernicke’s Encephalopathy: confusion, ophthalmoplegia, ataxia	50 – 100 mg/day from a B-complex supplement or high-potency multivitamin	Oral: 100 mg 2-3 times per day until symptoms resolve Intravenous: 200 mg three times daily, up to 500 mg twice daily for 3-5 days, then 250 mg/day for another 3-5 days or until symptoms resolve, then 100 mg/day oral indefinitely Intramuscular: 250 mg/day for 3-5 days or 100–250 mg/month
Vitamin B_2_ (Riboflavin) [30,26]	Not widely reported	Not widely reported	Cheilitis, glossitis, stomatitis, anemia, dermatitis	No specific dosing post-bariatric surgery has been established. Take as part of multivitamin tablets (take 1-2 tablets/day)	5 – 10 mg/day
Vitamin B_3_ (Niacin) [30,36]	Not widely reported	Not widely reported	Pellagra: dermatitis, diarrhea, dementia, glossitis, encephalopathy	No specific dosing post-bariatric surgery has been established. Take as part of multivitamin tablets (take 1-2 tablets/day)	100 – 500 mg three times daily
Vitamin B_5_ (Pantothenic Acid) [30,36]	Not widely reported	Not widely reported	Adrenal insufficiency, alopecia, dermatitis	No specific dosing post-bariatric surgery has been established. Take as part of multivitamin tablets (take 1-2 tablets/day)	2 – 4 d/day
Vitamin B_6_ (Pyridoxine) [30,36]	Not widely reported	Not widely reported	Sideroblastic anemia, glossitis, peripheral neuropathy, seizures, seborrheic dermatitis	No specific dosing post-bariatric surgery has been established. Take as part of multivitamin tablets (take 1-2 tablets/day)	30 mg/day
Vitamin B_7_ (Biotin) [30,36]	Not widely reported	Not widely reported	Alopecia, dermatitis, myalgia, lethargy, depression	No specific dosing post-bariatric surgery has been established. Take as part of multivitamin tablets (take 1-2 tablets/day)	20 mg/day
Vitamin B_9_ (Folic Acid) [2,25,36,40]	0% - 63.2%	9-39%	Megaloblastic anemia, glossitis, increased risk for neural tube defects	General population: 400 – 800 mg/day Women of childbearing age: 800 – 1000 mg/day	1000 mg/day until normal levels, then resume maintenance dosing
Vitamin B_12_ (Cobalamin) [2,36-40]	4.7 - 34.4%	4-20%	Megaloblastic anemia, peripheral neuropathy, reversible dementia, subacute degeneration of the cord	Oral: 350 – 1000 mg/day Nasal spray: as directed Parenteral (IM or SQ): 1000 mg/month	1000 mg/day until levels are normal then resume maintenance dosing
Calcium [25,26,36-40]	0.9%	1-25%	Myalgia, spasms, tetany, seizures	Should be taken in divided doses BPD/DS: 1800 – 2400 mg/day LAGB, SG, and RYGB: 1200 – 1500 mg/day	BPD/DS: 1800 – 2400 mg/day LAGB, SG and RYGB: 1200 – 1500 mg/day
Iron [31,36-38,42]	26.2%	33-49%	Microcytic anemia, koilonychia, pica, cheilitis, glossitis	Oral replacement should be taken in divided doses. Males and patients with no history of anemia: 18 mg/day Menstruating females or patients who had RYGB, SG or BPD/DS: 45 – 60 mg/day	Oral: 150 – 200 mg/day to as much as 300 mg 2-3 times daily Intravenous iron can be given if oral replacement is insufficient or not tolerated
Copper [25,36-39]	Not widely reported	10-15%	Depigmentation of skin, muscle weakness, osteoporosis, ataxia	BPD/DS and RYGB: 2 mg/day SG and LAGB: 1 mg/day Note: a ratio of 8-15 mg of zinc per 1 mg of copper should be given to minimize risk of copper deficiency	Mild-moderate: 3 – 8 mg/day oral copper gluconate or sulfate until levels are normal Severe: 2 – 4 mg/day intravenously for 6 days or until levels are normal
Selenium [25,30,36]	Not widely reported	11-46%	Cardiomyopathy	No specific dosing post-bariatric surgery has been established. Take as part of multivitamin tablets (take 1-2 tablets/day)	100 mg/day
Zinc [25,36-40]	Not widely reported	42-65% [25]	Alopecia, diarrhea, dermatitis, impaired wound healing, immune dysfunction	BPD/DS: 16 – 22 mg/day RYGB: 8 – 22 mg/day SG and LAGB: 8 – 11 mg/day Note: a ratio of 8-15 mg of zinc per 1 mg of copper should be given to minimize risk of copper deficiency	Insufficient evidence for a defined dose. Repletion dose should be determined based on routine monitoring of zinc levels. Some evidence to suggest benefit with 220 mg/day orally or 8 – 15 mg/day intravenously

Strategies to prevent nutritional deficiencies before and after bariatric surgeries

Research has clearly shown that individuals with obesity have a higher prevalence of nutritional deficiencies. Some studies found that 50% of patients with obesity will at least have one nutritional deficiency [41].

Some factors increasing the risk of developing deficiencies include low income and educational level, and poor general nutrition knowledge. Combined with reduced access to healthcare and the inability to afford appropriate supplements postoperatively, these factors make deficiencies particularly difficult to correct. Early identification of patients affected by these challenges and appropriate education on the importance of adherence to recommended nutritional supplementation, including signs and symptoms that may require immediate medical attention, is key to preventing complications [43,44].

**Figure 4:**
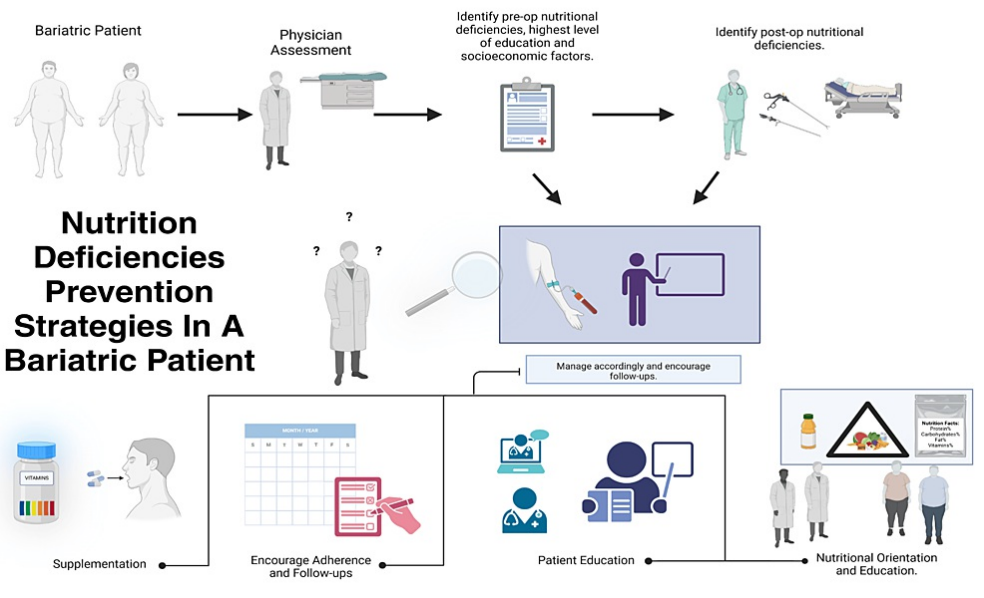
Strategies to prevent nutritional deficiencies in bariatric surgery patients. Image credits: Alberto Ayala Aguilar, 2024 (created with BioRender.com)

Despite appropriate nutritional supplementation, nearly 25% of postoperative patients develop micronutrient deficiencies [41]. Positive postoperative outcomes depend on identifying gaps in patients’ knowledge regarding diet and disease, the importance of developing good nutritional habits, with particular attention to decoding food labels, adhering to recommended supplementation, and providing appropriate preoperative orientation and education about the surgical procedure (see Figure 4) [43,45,46].

Decreased attendance at postoperative appointments within the first year has been associated with an increased risk of severe nutritional deficiencies and, therefore, increased mortality. A multicenter cross-sectional study funded by the Ontario Bariatric Network found that despite universal healthcare coverage, only 50% of the post-bariatric patients attended all of their follow-up appointments [44]. This data highlights the importance of encouraging long-term adherence to follow-up appointments, allowing monitoring and correction of patients’ micronutrient levels, and preventing deterioration of existing nutritional deficiencies or the development of new ones [44,47].

Treatment approaches for managing nutritional deficiencies in bariatric surgery patients 

Numerous strategies exist for addressing nutritional deficiencies in bariatric surgery patients. Pre- and postoperative dietary modifications and oral supplements are recommended. There is evidence that preoperative nutritional assessment and micronutrient correction may reduce postoperative deficiencies more effectively than no preoperative counseling [48]. In the initial 24-48 hours after surgery, a clear liquid diet (e.g., broth, herbal teas, diluted fruit juice) is advised to manage postoperative gastric edema. This gradually transitions to full liquids (milk, vegetable juice, yogurt, soup) over 10-14 days, followed by soft/creamy and then solid chewable foods over the next two to four weeks to minimize regurgitation and vomiting [49]. Emphasis is placed on chewing thoroughly before swallowing. To prevent hypoglycemia and dumping syndrome, it is recommended to consume smaller, frequent meals, avoid rapidly absorbable carbohydrates, and delay fluid intake to at least 30 minutes after meals [48,49]. Adhering to these guidelines can be difficult for patients for several reasons. Firstly, in countries where patients do not have universal healthcare, staying in hospital for two to three weeks postoperatively may not be financially feasible. Secondly, the prescribed diet is very restrictive, especially in the immediate postoperative period, and requires a lot of commitment to adhere to, which may be challenging once patients are discharged. 

Protein malnutrition is a common macronutrient deficiency that develops after bariatric surgery, necessitating protein intake of at least 60 g/day and potentially up to 1.5-2.1 g/kg of ideal body weight per day to preserve muscle mass [31,40,48,50,51]. Liquid protein supplementation is advised due to challenges in meeting these targets with solid foods [48]. Initial daily multivitamin supplementation (at least two chewable tablets per day for the first three to six months) is recommended to address common micronutrient deficiencies post-bariatric surgery (see Table 1 for prevention and treatment of specific deficiencies) [16,48]. Due to individual differences, the level of absorption of micronutrients is considerably varied, particularly with oral iron replacements [52]. Despite the recommended doses for postoperative micronutrient supplementation, regular blood chemistry monitoring is imperative for individualized adjustments [48,53,54]. Monitoring includes complete blood count, liver tests, glucose, electrolytes, creatinine, iron, vitamin B12, folate, calcium, parathyroid hormone, and 25-hydroxyvitamin D at three- to six-month intervals for the first two years postoperatively, as well as regular monitoring of albumin, vitamin A and zinc levels. After the first 24 months, blood tests should be monitored annually. Bone mineral density assessments are also recommended at 12-24 months, then two to five years thereafter [30,50,55,56]. Persistent, severe, or symptomatic deficiencies may require hospitalization and intramuscular or intravenous replacement [30]. A randomized controlled trial demonstrated the benefits of preoperative vitamin D supplementation, postoperative aerobic exercise, and continued vitamin D, calcium, and protein supplementation in preserving bone mineral density and lean body mass [57]. Dietician counseling pre- and postoperatively can promote healthy eating habits emphasizing nutritionally dense diets [48]. 

In cases where conservative treatments fail to meet nutritional needs, gastrostomy tubes (enteral nutrition) or total parenteral nutrition may be considered [49]. It is postulated that a slow adaptation process begins in the gastrointestinal system after bariatric surgery, which leads to increased expression of channel proteins responsible for micronutrient absorption, thereby reducing the need for supplementation over time [40].

Global disparities in addressing nutritional deficiencies after bariatric surgeries

In high-income countries, preventing and treating nutritional deficiencies following bariatric surgery is a concerted effort requiring coordinated care by multiple health professionals. Preventing such outcomes begins even before the operation. Aside from preoperative nutrient screening and correction by the bariatric multidisciplinary team (MDT), including registered dieticians, patients scheduled for surgery also undergo an extensive psychological, social, and behavioral evaluation by a behavioral health specialist to assess their ability to implement nutritional and behavioral changes to their diet before and after the procedure [37]. Following surgery, patients have regular follow-ups to monitor their progress and correct any micronutrient deficiency that may arise. Some guidelines have recommended life-long surveillance [56]. In contrast, others suggest routine monitoring can be discontinued after five years, partly due to postoperative intestinal adaptation, which may reduce the need for supplements over time [37].

One challenge high-income countries face is the availability of a wide range of bariatric supplements, some of which may not be approved by the Food and Drug Administration (FDA). This may cause micronutrient deficiencies in patients who are otherwise adhering to their prescribed postoperative nutritional plans. ASMBS recommends discussing such commercial products with patients at each visit to identify and prevent micronutrient deficiency early [37].

Another barrier to preventing postoperative nutritional deficiencies, not exclusive to high-income countries, is the cost of long-term nutritional supplements and frequent postoperative lab tests for monitoring. A study done in 2008 in Switzerland estimated the monthly cost of supplements to be $35 on average two years after a gastric bypass [58]. As this estimate may be significantly higher now due to inflation, this constitutes a real barrier to supplement compliance. There is a disproportionately higher prevalence of obesity affecting individuals of low socioeconomic and ethnic minority backgrounds, particularly within developed countries [59,60]. Factors contributing to this disparity include limited access to healthy foods and healthcare services or gyms [59]. In the United States, patients who are male, black, or Hispanic, and from a low socioeconomic background, are less likely to undergo bariatric surgeries [60]. 

In LMICs, on the other hand, patients face significant challenges in accessing healthcare, with 94% lacking access to basic surgical care compared to 14.9% in high-income countries [61]. As an example, a recent study on cardiac surgeries in LMICs reported that LMICs have approximately 0.04 adult cardiac surgeons per million of the population, compared to the 7.15 adult cardiac surgeons per million of the population in high-income countries [62]. However, improving access to medical care and bariatric surgery, without implementing a strategy to improve eating habits and levels of physical activity, is unlikely to lead to lasting changes [63]. A study conducted in the Middle Eastern region highlighted the scarcity of dedicated bariatric MDTs, including dieticians, bariatric physicians, endocrinologists, and psychologists, to help prepare patients for better intraoperative outcomes and follow patients postoperatively to ensure appropriate recovery, as these teams can be expensive to set up and maintain [64]. Other postoperative follow-up barriers in LMICs include transportation issues, particularly for rural residents, and limited communication with healthcare providers in case of complications or emergencies [65]. Furthermore, patients from LMICs have restricted financial and logistical access to prophylactic care, including nutrient supplementation to prevent deficiencies, compared to high-income countries [66]. This is made worse by comparatively low budget allocations towards the management of non-communicable diseases in LMICs compared to communicable ones [67].

This prominent inequity in access to healthcare between LMICs and high-income countries permeates beyond the management of obesity-related illnesses. While the focus of healthcare services in LMICs is on the prevention and treatment of infectious diseases, updated research warns of the increasing rates of morbidity and mortality in LMICs associated with preventable non-communicable diseases, particularly due to cardiovascular and chronic respiratory diseases [68-71]. LMICs account for approximately 85-90% of global premature deaths resulting from non-communicable diseases among 30-69 year olds [71]. Due to the cost associated with screening for and diagnosing diseases in their early stages, many preventable diseases are underdiagnosed or misdiagnosed [67,69]. Additionally, the epidemiological data collected from LMICs is inadequate and lacks proper disease surveillance [67,69,71]. As a result, public health policymakers and stakeholders do not always have accurate information on the true burden of these diseases and may therefore fail to allocate sufficient resources to address them [67]. 

Strategies to promote equity in nutritional deficiency prevention and treatment 

Access to bariatric surgery for obesity treatment highlights significant global disparities in healthcare resources and equitable provision. Ethnicity, age, socioeconomic status, and geographic location are crucial in determining access to this treatment. Alarmingly, those who could benefit the most - older individuals, racial or ethnic minorities, and patients with low income and education - are often the least likely to receive it, reflecting deep-seated systemic biases and inequalities within healthcare systems [72]. 

In high-income countries, addressing the prevention and treatment of nutritional deficiencies necessitates a multifaceted approach to ensure equity across diverse population groups. This encompasses identifying and rectifying disparities in access to nutritional services and addressing underlying systemic barriers that hinder the equitable provision of care. Enhancing accessibility to services and empowering individuals to manage their health effectively are important steps in narrowing the gap in nutritional health outcomes [73]. Additionally, it is crucial to train healthcare professionals to navigate social disparities in patient care effectively. Training should focus on cultural competency, sensitivity to socioeconomic factors, and communication strategies to ensure patient-centered care for all individuals [74].

Due to the challenges in implementing surgical treatments for obesity in LMICs, efforts should focus more on obesity prevention. Emphasis should be on identifying and addressing poor childhood nutrition [63] as this is a predictor for adult obesity; the provision of healthy, locally sourced foods in public institutions, and emphasis on nutrition education in schools can improve food literacy, encouraging the development of healthy food habits. Furthermore, public health campaigns that dismantle cultural stigmas that associate healthy weights with HIV/AIDS illness and provide comprehensive education to communities on the long-term debilitating effects of obesity, may support a cultural shift away from glorifying obesity. On a government level, subsidizing nutritious foods and taxing heavily processed, unhealthy foods may help to decrease the prevalence of obesity in the population [19].

Battling the existing obesity crisis in LMICs will require increased training of local healthcare professionals, aiming to create regional specialist centers with a dedicated MDT specializing in bariatric pre- and postoperative care. Previous models consisting of experienced and retired surgeons taking sabbatical years to work collaboratively with and train local professionals have shown success [62[. Furthermore, many LMICs are already working to implement National Surgical, Obstetric, and Anesthesia Plans (NSOAPs) to strengthen their healthcare systems. NSOAPs focus on the improvement of infrastructure, workforce, service delivery, financing, governance, and information management. Many countries in sub-Saharan Africa have already begun implementing these plans [68]. Financing such endeavors needs innovative solutions - at a local level, some centers have been successful in employing a co-financing model where patients with higher incomes pay a higher fee, to allow lower-income patients to receive treatment at a reduced cost [62]. At an international level, the responsibility lies with healthcare professionals to advocate to their governments to expand the provision and development of specialized surgical services, with dedicated MDTs, in LMICs. Finally, more surveillance and research need to be conducted on the prevalence, prevention, and treatment of non-communicable diseases in LMICs. In order to allow equitable allocation of resources, rigorous systematic reviews [75,76] focusing on the socioeconomic disparities impacting the management of nutritional deficiencies in bariatric surgery patients are required.

## Conclusions

In conclusion, bariatric surgery continues to be a pivotal intervention for morbid obesity, offering significant benefits including substantial weight loss and improvement of obesity-associated comorbidities. However, this review underscores the critical challenge of managing nutritional deficiencies pre- and postoperatively, highlighting the necessity of preoperative nutritional assessments and adherence to postoperative supplementation. The disparity in healthcare access between low, medium, and high-income countries exacerbates these challenges, underscoring the need for international collaboration to ensure equitable access to bariatric care and nutritional support. Strategies to address these disparities include training of appropriate healthcare professionals that form the bariatric MDT, enhancing patient education, ensuring consistent follow-up care, and adopting a multidisciplinary approach to patient management. This comprehensive review advocates for a multifaceted strategy to prevent and manage nutritional deficiencies, emphasizing the importance of equitable healthcare practices to improve outcomes and access to care for all bariatric surgery patients.
